# Einfluss kardiovaskulärer Erkrankungen auf die Fahreignung: Mobilitätseinschränkungen im Alter

**DOI:** 10.1007/s00103-024-03912-7

**Published:** 2024-06-21

**Authors:** Jan Rieß, Niklas Schenker, Fabian J. Brunner, Tobias Tönnis

**Affiliations:** Universitäres Herz- und Gefäßzentrum, Universitätsklinikum Hamburg-Eppendorf, Martinistr. 52, 20246 Hamburg, Deutschland

**Keywords:** Fahreignung, Fahrtauglichkeit, Mobilität, Privatfahrer:innen, Kardiologie, Driving fitness, Mobility, Private driver, Cardiology, Fitness to drive

## Abstract

Die Prävalenz von Herz-Kreislauf-Erkrankungen steigt mit zunehmenden Alter an. Häufige Symptome sind Atemnot, Brustschmerzen, Schwindel oder Synkopen, welche die Fahreignung beeinflussen können. Aufgrund einer zunehmenden Anzahl an Privatfahrer:innen über 65 Jahren und einer steigenden Prävalenz kardiovaskulärer Erkrankungen rücken Fragestellungen zur Einschränkung der Fahreignung von kardiologischen Patient:innen zunehmend in den klinischen Vordergrund. Dieser Artikel soll aktuelle Empfehlungen zur Fahreignung im Kontext kardiovaskulärer Erkrankungen zusammenfassen. Die Grundlage der Vorgaben stellen die Anlage 4 der Fahrerlaubnisverordnung, die Begutachtungsleitlinie der Bundesanstalt für Straßenwesen sowie die Leitlinien der Deutschen Gesellschaft für Kardiologie zur Fahreignung dar. Originalliteratur zu diesem Thema ist nur begrenzt vorhanden.

Mit Betonung auf eine individualisierte Bewertung werden klare Vorgaben für die Fahreignung bei kardialen Erkrankungen bzw. deren Symptomen oder Behandlungen formuliert. Die resultierenden Beeinträchtigungen können von wenigen Wochen bis hin zur generellen Aufhebung einer Fahreignung ausfallen. Führenden Einfluss auf die Bewertung der Fahreignung nehmen unabhängig von der kardialen Erkrankung die Symptomatik und die Wahrscheinlichkeit für einen plötzlichen Bewusstseinsverlust ein. Regelmäßige Untersuchungen sowie differenzierte Beurteilungen durch medizinisches Fachpersonal sind Voraussetzung für den Erhalt der Fahreignung.

Die Fahreignung älterer Privatfahrer:innen stellt ein bedeutendes und praxisrelevantes Thema in der Kardiologie dar. Aktuelle Richtlinien unterstützen die behandelnden Ärzt:innen entsprechende Empfehlungen auszusprechen.

## Abstract (en)

The prevalence of cardiovascular diseases increases with age. Common symptoms such as dyspnea, chest pain, dizziness, or syncope can impact driving fitness. Due to a growing number of private drivers aged 65 and older and an increasing prevalence of cardiovascular diseases, questions regarding driving fitness restrictions for cardiological patients are gaining prominence in clinical settings. This article aims to summarize current recommendations for driving fitness in the context of cardiovascular diseases. The basis for the guidelines includes the Driving License Ordinance, the expert assessment guidelines of the Federal Highway Research Institute, and the guidelines of the German Society of Cardiology on driving fitness. Original literature on this topic is limited.

Emphasizing an individualized assessment, clear guidelines for driving fitness in cardiac diseases or their symptoms and treatments are formulated. Regardless of the cardiac condition, the symptoms and likelihood of sudden loss of consciousness play a leading role in driving fitness assessment. Resulting impairments can range from a few weeks to a complete revocation of driving fitness. Regular examinations and differentiated assessments by medical professionals are prerequisites for maintaining driving fitness.

The driving fitness of older private drivers is a significant and practical topic in cardiology. Current guidelines support the treating physicians in providing appropriate recommendations.

## Einführung

Mobilität bedeutet Selbstständigkeit und Freiheit, sie ist ein wichtiger Faktor für Lebensqualität. Ältere Menschen müssen ihr Mobilitätsverhalten in der Regel schrittweise ändern und an ihre Fähigkeiten anpassen. Aber gerade für sie ist es wichtig, mobil zu sein, um ihr soziales Netzwerk zu pflegen und am gesellschaftlichen Leben teilhaben zu können.

Kardiovaskuläre Erkrankungen wie die koronare Herzkrankheit, Herzklappenerkrankungen, Herzrhythmusstörungen und die Herzinsuffizienz gehören zu den wichtigsten Ursachen für Morbidität und Mortalität im Alter. Zudem ist das Lebensalter an sich ein bedeutender kardiovaskulärer Risikofaktor, der eine Zunahme von anderen Komorbiditäten wie der arteriellen Hypertonie und des Diabetes mellitus mit sich bringt. Die altersspezifische Lebenszeitprävalenz einer „bedeutsamen kardiovaskulären Erkrankung“ steigt mit dem Alter und beträgt in der Altersgruppe ≥80 Jahre 45,1 % [1].

In diesem Artikel werden Einschränkungen der Fahreignung durch Herz-Kreislauf-Erkrankungen für Privatpersonen (im Folgenden „Privatfahrer:innen“) näher beleuchtet. Dabei gelten diese Einschränkungen altersunabhängig, betreffen aber Ältere in erhöhtem Maße. Regelungen für Berufsfahrer:innen sind gesondert zu treffen und werden in dem vorliegenden Artikel nicht behandelt. Maßgeblich für die Entscheidung, ob jemand ein Auto führen darf, ist das Risiko, einen Unfall zu verursachen. Dies ist grundsätzlich eng mit dem Risiko für das Auftreten von plötzlichen Bewusstseinseinschränkungen oder Synkopen verbunden. Die Grundlage zur Einschätzung des individuellen Risikos bietet die „Risk of Harm Formula“ der kanadischen Gesellschaft für Kardiologie [2]. Diese berücksichtigt verschiedene Kriterien:

- kumulative Zeit hinter dem Steuer,
- die Art des gefahrenen Fahrzeugs,
- Risiko für plötzlichen Kontrollverlust aufgrund von Herz-Kreislauf-Problemen,
- Risiko für Schwerverletzte oder Tote bei einem Autounfall.

Ärzt:innen sollen und dürfen nicht melden, ob eine eingeschränkte Fahrtauglichkeit vorliegt, da sie an die ärztliche Schweigepflicht gebunden sind. Sie sollten aber eine klare Empfehlung gegenüber ihren Patient:innen aussprechen und dies auch dokumentieren. Die oben genannten Kriterien bieten die Grundlage für die Empfehlungen, die durch Fachärzt:innen abgegeben werden.

## Fahreignung bei koronarer Herzkrankheit (KHK)

Zur Einschätzung der Fahreignung bei koronarer Herzerkrankung (KHK) wird unterschieden zwischen (1) einer stabilen Angina pectoris bzw. dem chronischen Koronarsyndrom und (2) dem Herzinfarkt bzw. dem akuten Koronarsyndrom.

Eine *chronisches Koronarsyndrom* beschreibt eine Form von Brustschmerzen, oder im Einzelfall auch belastungsabhängige Luftnot, welche unter körperlicher oder emotionaler Belastung auftritt und in Ruhe wieder sistiert. Diese Beschwerden werden durch eine vorübergehende Minderdurchblutung des Herzens verursacht. Unter dem *akuten Koronarsyndrom* wird eine Gruppe von akut einsetzenden Durchblutungsstörungen des Herzens verstanden. Diese umfassen die instabile Angina pectoris und den Myokardinfarkt, inkl. des ST-Strecken-Hebungs-Infarktes (STEMI) und des Nicht-ST-Strecken-Hebungs-Infarktes (NSTEMI). Hierbei treten die Symptome in der Regel plötzlich auf bzw. es tritt eine Verschlechterung des bekannten chronischen Zustandes ein [3]. Neben typischen anfallsartigen Brustschmerzen und teilweise Ausstrahlungen in den Arm, den Kiefer, den Rücken oder den Oberbauch können Symptome wie Atemnot, Übelkeit, Erbrechen, Schwindel oder Schweißausbrüche auftreten.

Die Beurteilung der Fahreignung richtet sich in erster Linie nach der Symptomatik:

### Chronisches Koronarsyndrom

*Asymptomatische KHK:* Eine asymptomatische koronare Herzkrankheit beeinflusst die Fahreignung von Patient:innen in der Regel nicht [4].

*Stabile Angina pectoris:* Patient:innen mit *stabiler Angina pectoris *unterliegen in der Regel ebenfalls keinen Restriktionen hinsichtlich ihrer Fahreignung. Zur individuellen Einschätzung sowie Verlaufsbeurteilung sind regelmäßige fachärztliche Untersuchungen erforderlich [4].

*Perkutane Koronarintervention:* Bei gutem klinischen Ergebnis beeinflusst eine etwaige kathetergestützte perkutane Koronarintervention (PCI) die Fahreignung nicht. Gegebenenfalls sind insbesondere im direkten Anschluss an einen Eingriff weitere Faktoren zu berücksichtigen, die eine aktive Teilnahme am Straßenverkehr vorübergehend einschränken (z. B. die Gabe von Sedativa; [5]).

*Bypass-Operation:* Sollte zur Verbesserung der koronaren Durchblutung alternativ eine Bypass-Operation durchgeführt werden, kann von einem Wiedererlangen der Fahreignung erst nach 2–4 Wochen ausgegangen werden. Die individuelle Einschätzung ist unter anderem abhängig von der individuellen Rehabilitation und erfordert in der Regel eine fachärztliche Untersuchung [4].

### Akutes Koronarsyndrom

Das *akute Koronarsyndrom* (ACS) umfasst sowohl die *instabile Angina pectoris* als auch den *akuten Myokardinfarkt*. Individuell berücksichtigt werden sollte die Tatsache, dass sowohl während des Akutereignisses als auch in den Tagen danach das Risiko für das Auftreten lebensbedrohlicher Herzrhythmusstörungen erhöht ist [6]. In der Regel befinden sich die Patient:innen in den Tagen nach dem Akutereignis weiterhin in stationärer Überwachung, um eben solche Herzrhythmusstörungen frühzeitig erkennen zu können. Entscheidend für die Fahreignung nach Entlassung ist insbesondere eine mögliche Funktionseinschränkung der linksventrikulären (LV-)Funktion. Liegt die LV-Funktion ≤35 % besteht eine Fahreignung erst nach 4 Wochen und zuvor durchgeführter fachärztlicher Untersuchung. Liegt die LV-Funktion >35 % wird im Falle eines komplikationslosen stationären Verlaufs nach ACS die Fahreignung nach dem Klinikaufenthalt als gegeben angesehen. Sollten sich im Rahmen des ACS eine akute kardiale Dekompensation und eine Verschlechterung einer Herzinsuffizienz zeigen, so gilt die 4‑wöchige Restriktion unabhängig von der LV-Funktion [5, 7].

## Fahreignung bei arterieller Hypertonie

Die arterielle Hypertonie (Bluthochdruck) gehört zu den häufigsten Erkrankungen des kardiovaskulären Systems in Deutschland [8]. Die Prävalenz steigt mit zunehmenden Alter auf 70 % bei Patient:innen >65 Jahre [9]. Häufig verläuft eine arterielle Hypertonie bzw. eine unzureichende Blutdruckeinstellung asymptomatisch. Allerdings können in Einzelfällen sowohl besonders hohe Blutdruckwerte als auch ein rascher Blutdruckabfall (z. B. nach Medikamenteneinnahme) mit Symptomen einhergehen, die die Fahreignung beeinflussen können. Typische Symptome sind häufig zerebraler Natur (Kopfschmerzen, Schwindel, Sehstörungen, Benommenheit etc.) oder präsentieren sich unspezifisch mit Herzrasen, Kurzatmigkeit und ggf. Angina pectoris.

*Asymptomatische arterielle Hypertonie:* Ein symptomlos erhöhter Blutdruck beeinflusst die Fahreignung von Privatfahrer:innen nicht [4].

*Symptomatische arterielle Hypertonie:* Bei Privatfahrer:innen mit zerebralen Symptomen aufgrund eines Bluthochdrucks besteht *keine* Fahreignung, unabhängig von den gemessenen Blutdruckwerten [4, 5].

*Neue Medikation:* Nach (neuer) Medikamenteneinnahme zur Behandlung der arteriellen Hypertonie besteht eine erhöhte Wahrscheinlichkeit eines Kontrollverlustes am Steuer aufgrund oben angegebener Symptome. Es sollte entsprechend eine individuelle Empfehlung nach fachärztlicher Untersuchung erfolgen [4].

## Fahreignung bei Herzklappenerkrankungen

Patient:innen mit Herzklappenerkrankungen können abhängig von deren Schweregrad und der betroffenen Herzklappe an unterschiedlichen Symptomen leiden. Das klinische Bild reicht von asymptomatischen Befunden bis hin zu starken klinischen Symptomen mit Zeichen der Herzinsuffizienz, Schwindel oder gar Synkopen. Nach Einschätzung der Autoren bedarf es bei der Beratung hinsichtlich einer Fahreignung bei vorliegender Erkrankung der Herzklappen einer zusätzlichen individuellen Betrachtungsweise der betroffenen Herzklappe. So gehen beispielsweise Insuffizienzen der Mitral- oder Trikuspidalklappe in der Regel mit Symptomen der Herzinsuffizienz wie Ödemen und Belastungsdyspnoe einher, die in einer Vielzahl der Fälle medikamentös eingestellt werden können und eine stabile Belastbarkeit der Patient:innen ermöglichen. Auf der anderen Seite kommen bei einer hochgradigen Aortenklappenstenose häufig neben einer Belastungseinschränkung mit klassischen Symptomen wie Dyspnoe und Angina pectoris auch Schwindel und (Prä‑)Synkopen vor, die somit im Einzelfall die Fahreignung weitaus stärker beeinflussen können. Insbesondere aufgrund der mit dem Alter zunehmenden Prävalenz der degenerativen Aortenklappenstenose sollte dies im Rahmen der fachärztlichen Untersuchung beachtet werden.

Grundsätzlich richtet sich die Bewertung einer individuellen Fahreignung nach dem klinischen Befund und ist im Folgenden aufgeführt.

*Asymptomatische (gering symptomatische) Herzklappenerkrankung: *Asymptomatische oder gering symptomatische Patient:innen mit Herzklappenerkrankungen erfahren in der Regel keine Einschränkung der Fahreignung [4, 5].

*Symptomatische Herzklappenerkrankungen:* Im Falle einer stärker ausgeprägten Symptomatik (z. B. Atemnot bereits in Ruhe) oder nach stattgehabter Synkope besteht *keine* Fahreignung. Diese kann erst nach erfolgreicher Behandlung des Herzklappenfehlers wiedererlangt werden [4, 5].

*Interventionelle/chirurgische Behandlung von Herzklappenerkrankungen: *Es gibt keine eindeutige Zeitvorgabe, ab wann nach erfolgter Klappentherapie eine Fahreignung wieder gegeben ist. Eine vollständige Rehabilitation nach dem Eingriff wird für eine Fahreignung vorausgesetzt. Beispielsweise in Australien gilt nach einem Herzklappeneingriff ein Fahrverbot für 4 Wochen [10].

## Fahreignung bei Synkopen

Synkopen sind kurzzeitige Zustände einer vollständigen Bewusstlosigkeit aufgrund vorübergehender Minderdurchblutung des Gehirns. Sie können neurologische oder kardiovaskuläre Ursachen haben. Eine gründliche Anamnese und ausführliche Diagnostik, unter anderem mittels Langzeit-EKG, ggf. der Implantation eines Ereignisrecorders, Blutdruckanalysen, neurologischer Untersuchung, HNO-ärztlicher Untersuchung oder neurovaskulären Ultraschalls, sind entscheidend, um eine Risikoabschätzung für das erneute Auftreten sinnvoll durchführen zu können.

*Erstmalige Synkope: *Im Allgemeinen gibt es nach einer ersten Synkope keine Einschränkungen der Fahreignung [4, 5], es sei denn, es liegen Hinweise auf ein sehr hohes (Rezidiv‑)Risiko (zum Beispiel hochgradige Aortenklappenstenose, schwere KHK, EKG-Veränderungen, wie beispielsweise ein höhergradiger atrioventrikulärer Block (AV-Block), bifaszikuläre Blockbilder) vor [11]. Hier besteht *keine* Fahreignung bis zur definitiven Therapie der zugrunde liegenden Erkrankung [5].

*Rezidivierende Synkopen: *Bei wiederholten (unklaren) Synkopen wird eine erneute Diagnostik empfohlen (Suche nach höhergradigen Herzrhythmusstörungen). Die Fahreignung wird frühestens nach 6 Monaten in Betracht gezogen. Dieser Zeitraum kann individuell angepasst werden. Es gibt Ausnahmefälle, in denen Einzelentscheidungen getroffen werden können, insbesondere wenn (auch durch erfolgte Therapien) die Wahrscheinlichkeit weiterer Synkopen gering ist. Beispiele hierfür sind die Miktions- oder auch die orthostatische Synkope [4, 5].

## Fahreignung bei bradykarden Arrhythmien

Der zu langsame und arrhythmische Herzschlag (bradykarde Arrhythmie) kann unter Umständen auch zum Auftreten von Synkopen führen. Ursächlich sind unter anderem Schädigungen des spezifischen Reizleitungssystems des Herzens (Sinusknoten, atrioventrikulärer Knoten, Tawara-Schenkel), die zum Beispiel durch einen Herzinfarkt verursacht werden oder auch iatrogen, also als Folge ärztlicher Eingriffe, entstehen können. Weiter kann es auch durch Überaktivität des parasympathischen Nervensystems oder als Folge von Einnahme einer bradykardisierenden Medikation zu einer deutlichen Abnahme der Herzfrequenz kommen.

*Bradykardie mit Bewusstseinseinschränkung: *Führen Bradykardien zu Synkopen und ist die Herzrhythmusstörung noch nicht therapiert, besteht *keine* Fahreignung. Dies gilt auch für AV-Blockierungen (siehe unten) mit konsekutiver Bewusstseinseinschränkung [4, 5].

*Sonderfall AV-Block: *Eine Blockierung innerhalb des Reizleitungssystems auf Höhe des sogenannten AV-Knotens ist bis zum AV-Block Grad IIb (Mobitz) nicht mit einem Fahrverbot assoziiert, sofern kein Bewusstseinsverlust vorlag. Lediglich ein erworbener AV-Block Grad III führt zum Verlust der Fahreignung, auch wenn er asymptomatisch ist. Jedoch kann nach einer effektiven Therapie (Medikamente/Schrittmachertherapie) und kardiologischer Nachuntersuchung die Fahreignung wiederhergestellt sein. Es ist zu beachten, dass nach einer Schrittmachertherapie die entsprechenden Auflagen (siehe unten) berücksichtigt werden müssen [4, 5].

## Fahreignung bei tachykarden supraventrikulären Arrhythmien

Diffiziler ist die Bewertung der Fahreignung bei tachykarden, supraventrikulären Arrhythmien (Herzrhythmusstörungen, die ihren Ursprung in den Herzvorhöfen haben), da hier nicht automatisch von einer Beeinträchtigung des Bewusstseins oder der Reaktivität ausgegangen werden kann. Unter dem Überbegriff der supraventrikulären Tachyarrhythmien wird hier eine heterogene Gruppe von Rhythmusstörungen zusammengefasst (Vorhofflimmern, Vorhofflattern, fokale atriale Tachykardien, kreisende Erregungen zwischen Vorhöfen und Hauptkammern). Eine tiefergreifende Einteilung wird in Bezug auf die Fahreignung nicht notwendig, da hier, wie auch bei den bradykarden Arrhythmien, eine Einteilung nach dem Auftreten von Synkopen erfolgt.

*Supraventrikuläre, tachykarde Arrhythmie mit (Prä)synkope: *Es besteht *keine* Fahreignung, wenn die ursächlichen supraventrikulären, tachykarden Herzrhythmusstörungen noch vorhanden und unbehandelt sind. Ist eine erfolgreiche Therapie, inklusive kardiologischer Nachuntersuchung, erfolgt, kann die Fahreignung wiedererlangt werden [4].

*Supraventrikuläre, tachykarde Arrhythmie ohne Bewusstseinseinschränkung:* Es besteht Fahreignung [5].

## Fahreignung bei ventrikulären Arrhythmien

Herzrhythmusstörungen aus den Hauptkammern des Herzens (ventrikuläre Arrhythmien) sind häufig Folge von strukturellen Schäden an der Herzmuskulatur, beispielsweise nach einem Myokardinfarkt. Nicht immer sind ventrikuläre Arrhythmien mit einem Bewusstseinsverlust assoziiert und nicht immer sind diese als maligne Herzrhythmusstörungen einzuordnen. So können auch bei strukturell unauffälligem Herzmuskel (nachgewiesen durch kardiale Bildgebung wie Echokardiographie oder Magnetresonanztomographie) Extraschläge aus bestimmten Regionen des Herzens entstehen, die nicht zur Beeinträchtigung des Bewusstseins führen.

Es wird generell zwischen Dauer und Genese der Arrhythmie (z. B. Kardiomyopathie, Ionenkanalerkrankungen, idiopathisch) unterschieden und auch hier spielt die hämodynamische Stabilität eine Rolle für die Empfehlungen in Bezug auf die Kraftfahreignung.

*Ventrikuläre Extrasystolen:* Extraschläge aus den Hauptkammern des Herzens sind häufig benigne und führen in der Regel nicht zum Bewusstseinsverlust. Sie haben daher keine Auswirkung auf die Fahreignung [5].

*Nicht anhaltende Kammertachykardie: *Eine nicht anhaltende ventrikuläre Tachykardie (nsVT, Dauer von ≤30 s) ohne symptomatische Beeinträchtigung führt nicht zu einer Einschränkung der Fahreignung. Bei Vorliegen von Symptomen muss nach kardiologischer Nachuntersuchung individuell über eine Fahreignung entschieden werden, abhängig von der Art der Symptomatik [5]. Hier wird unter anderem auch die Morphologie der Tachykardie (monomorph, polymorph) mit berücksichtigt.

*Anhaltende Kammertachykardie: *Anhaltende ventrikuläre Tachykardien (sVT, Dauer von >30 s) mit oder ohne Synkope führen nur dann nicht zum Verlust der Fahreignung, wenn die Arrhythmie effektiv behandelt und eine erfolgreiche Arrhythmiekontrolle (medikamentös oder interventionell, beispielsweise durch Katheterverödung) eingeleitet wurde [4, 5]. Es ist jedoch zu beachten, dass bei einer Indikation für einen implantierbaren Kardioverter-Defibrillator (ICD) die spezifischen ICD-Empfehlungen berücksichtigt werden müssen (siehe nächster Abschnitt).

## Fahreignung bei Schrittmacher und implantiertem Defibrillator (ICD)

Herzschrittmacher finden in der Medizin vor allem bei der Behandlung von bradykarden Herzrhythmusstörungen und der Vorbeugung von (rezidivierenden) Synkopen Anwendung. Darüber hinaus spielen spezielle Formen von Herzschrittmachern auch bei der Therapie der Herzinsuffizienz eine Rolle (kardiale Resynchronisationstherapie).

ICD werden zur Therapie der tachykarden Herzrhythmusstörungen verwendet und dienen primär dem Unterbrechen von ventrikulären Tachykardien und Kammerflimmern. Die Funktion der antitachykarden Überstimulation (ATP) erweitert bei den meisten Aggregaten die Therapieoptionen und ist bei guter Effektivität eine schonendere Behandlung. Die Indikationsstellung unterliegt einem steten Wandel, da neue Therapieformen, gerade bei der Behandlung der Herzschwäche, auch einen Einfluss auf die Auftretenswahrscheinlichkeit der Arrhythmien haben.

Man unterscheidet generell zwischen der primärprophylaktischen und der sekundärprophylaktischen Indikation für einen ICD, wobei Letztere nach stattgehabtem Arrhythmieereignis (mit oder ohne Synkope) oder überlebtem plötzlichen Herztod (PHT) erfolgt. Patient:innen mit sekundärprophylaktischer ICD-Implantation weisen insgesamt eine höhere Rate an Arrhythmien auf. Wichtig ist hier zu betonen, dass nicht das Aggregat selbst, sondern die zugrunde liegende Herzerkrankung ausschlaggebend für die Kraftfahreignung ist.

*Herzschrittmacher: *Nach einer Schrittmacherimplantation oder einem Aggregatwechsel (beispielsweise bei Dysfunktion oder Batterieerschöpfung) ist die Fahreignung in der Regel gegeben, sofern die regelrechte Funktion überprüft und die Wundheilung abgeschlossen ist. Es ist jedoch wichtig, regelmäßige kardiologische Kontrolluntersuchungen durchzuführen, um die Sicherheit im Straßenverkehr zu gewährleisten [4, 5].

*ICD: *Nach einer primärpräventiven ICD-Implantation besteht die Fahreignung bereits nach 1–2 Wochen, sofern regelmäßige kardiologische Kontrollen (inkl. ICD-Überprüfungen) erfolgen. Bei Ablehnung des ICD besteht in der Regel weiterhin Fahreignung. Für die Sekundärprävention ist die Fahreignung bei adäquater ICD-Funktion frühestens 3 Monate nach Ereignis gegeben, dies gilt auch hier ebenfalls bei Ablehnung des ICD, sofern keine weiteren Rhythmusereignisse aufgetreten sind. Nach adäquater Schockabgabe (Schockabgabe, die auf eine korrekt erkannte Tachykardie folgt) kann eine Fahreignung frühestens nach 3 Monaten gegeben sein, nach inadäquater Schockabgabe (Schockabgabe, die auf eine fälschlicherweise als Tachykardie erkannte Episode/auf Störartefakte oder aufgrund von ICD-Fehlfunktionen folgt) ist die Fahreignung dann gegeben, wenn die zugrunde liegende Ursache erkannt und beseitigt wurde [4, 5].

*Aggregat- oder Sondenwechsel: *Nach einem routinemäßigen Aggregat- oder Sondenwechsel ist die Fahreignung nach 1–2 Wochen gegeben [5].

*Elektrischer Sturm:* Der elektrische Sturm (definiert als 3 oder mehr sVT in 24 h) ist ein Einzelfall und bedarf sorgfältiger kardiologischer Aufarbeitung [4, 5]. Zur Abklärung der Fahreignung kann eine spezielle rhythmologische Untersuchung (elektrophysiologische Untersuchung) notwendig sein.

Interessant ist an dieser Stelle auch ein kurzer Blick in andere Länder. So führt zum Beispiel in Kanada eine inadäquate Schockabgabe nicht zur Aberkennung der Fahreignung und nach Aggregatwechsel wird eine Fahreignung (ohne die Angabe einer minimalen Zeitspanne) ausgesprochen, sofern die „Erholung“ vom jeweiligen Eingriff abgeschlossen ist [12]. Im Vereinigtem Königreich führt dagegen beispielsweise eine ICD-Implantation aufgrund einer anhaltenden Kammertachykardie mit Bewusstseinseinschränkung zum Verlust der Fahreignung für 6 (statt wie in Deutschland für 3) Monate [13]. Im Allgemeinen sind die Empfehlungen der verschiedenen Länder jedoch überwiegend deckungsgleich.

## Fahreignung bei Ionenkanalerkrankungen

Ionenkanalerkrankungen sind Erbkrankheiten, die durch Mutationen der entsprechenden Gene zu abnormalen elektrischen Signalen in den Zellen führen. Die Folge sind unter anderem Herzrhythmusstörungen, Muskelschwäche oder neurologische Symptome. Nicht selten ist der PHT das erste Symptom der Erkrankungen, die insgesamt eine niedrige Prävalenz aufweisen. In Bezug auf kardiale Arrhythmien spielen das Long-QT-Syndrom, das Brugada-Syndrom (BrS), das Short-QT-Syndrom und die katecholaminerge polymorphe ventrikuläre Tachykardie (CPVT) die größte Rolle [14].

*Long-QT-Syndrom:* Asymptomatische Personen mit der Diagnose Long-QT-Syndrom, die diese aufgrund von Familienuntersuchungen erhalten haben, sind in der Regel fahrtauglich. Dies ist jedoch *nicht* der Fall, wenn Synkopen, Torsades-des-Pointes-Tachykardien („Spitzenumkehrtachykardien“) oder eine QTc-Zeit (spezieller Abschnitt des Herzzyklus im EKG) von mehr als 500 Millisekunden vorliegen. Die Therapie besteht hier vorwiegend in der Prävention des PHT mittels ICD und der Medikation mit Betablockern. Nach Einleitung einer Therapie kann ggf. nach kardiologischen Nachkontrollen eine Fahreignung wieder gegeben sein [4, 5]. Nach Implantation eines ICDs sind die entsprechenden Auflagen (siehe oben) zu berücksichtigen.

*Brugada-Syndrom: *Auch Personen mit Diagnose eines BrS, die diese zufällig (im engeren Sinne entspricht dies zunächst lediglich einem Brugada-EKG, die Diagnose eines BrS kann ohne Symptomatik oder genetische Untersuchung nicht gestellt werden) oder im Rahmen von Familienuntersuchungen erhalten haben, sind fahrtauglich. Dies gilt nicht nach einem überlebten PHT [4, 5]. Bei fehlendem Nachweis der rhythmogenen Genese einer Synkope ist bei lediglich medikamentös provoziertem Brugada-EKG nicht von einem deutlich erhöhten Risiko für einen PHT auszugehen [11]. Insgesamt muss hier nach kardiologischer Untersuchung im Einzelfall über die Fahrtauglichkeit entschieden werden und es gelten die Empfehlungen für Patient:innen mit Synkope [5]. Nach Implantation eines primär- oder sekundärprophylaktischen ICDs bestehen die bereits genannten Einschränkungen (siehe oben).

*Short-QT-Syndrom/CPVT:* Bei Vorliegen eines Short-QT-Syndroms oder einer CPVT besteht Fahreignung je nach Situation. Hier wird in kardiologischen Untersuchungen wie in den vorangegangenen Abschnitten vor allem das Auftreten von Bewusstseinseinschränkungen oder das Überleben eines PHTs sowie das Vorhandensein eines ICDs bewertet [4].

## Fahreignung bei Herzinsuffizienz

Bei Patient:innen mit Herzinsuffizienz beruht die Beurteilung der Kraftfahreignung bei Privatfahrer:innen in der Regel auf der klinischen Symptomatik. Krankheitsursache und gemessene Herzleistung spielen eine untergeordnete Rolle. Die Stratifizierung erfolgt gemäß der internationalen Funktionsklassifikation der NYHA (New York Heart Association [15]; Tab. 1). Die Therapie der Herzinsuffizienz hat sich in den letzten Jahrzehnten signifikant verbessert und kann einen großen Einfluss auf die Prognose nehmen [16]. Die individuelle Therapietreue (Compliance) der Patient:innen sollte daher neben der klinischen Symptomatik ebenfalls bei der Prüfung der Fahreignung berücksichtigt werden [4].

**Tab. 1:** Funktionsklassifikation der NYHA (New York Heart Association) zur Einteilung der Herzinsuffizienz [15]

NYHA-Klasse I	Keine Einschränkungen der körperlichen Aktivität. Normale körperliche Aktivität verursacht keine ungewöhnliche Erschöpfung oder Atemnot
NYHA-Klasse II	Geringfügige Einschränkungen der körperlichen Aktivität. Die Patient:innen sind bei normaler körperlicher Aktivität belastet, erleben jedoch keine Symptome in Ruhe
NYHA-Klasse III	Merkliche Einschränkungen der körperlichen Aktivität. Die Patient:innen sind bei geringer körperlicher Aktivität eingeschränkt und erleben Symptome wie Erschöpfung, Herzrasen oder Atemnot. In Ruhe treten jedoch keine Symptome auf
NYHA-Klasse IV	Starke Einschränkungen der körperlichen Aktivität. Die Patient:innen erleben bereits bei geringer körperlicher Aktivität starke Symptome und haben auch in Ruhe Beschwerden

*Geringe Symptomatik: *Eine Herzinsuffizienz der NYHA-Klasse I oder II beeinflusst die Fahreignung der Privatfahrer:innen in der Regel nicht [4, 5].

*Deutliche Symptomatik:* Bei Patient:innen mit einer Herzinsuffizienz in der NYHA-Klasse III ist die Fahreignung nur bei einem stabilen Verlauf der Herzinsuffizienz nach fachärztlicher Untersuchung gegeben [4, 5].

*Starke Symptomatik:* Patient:innen der NYHA-Klasse IV sowie mit instabiler NYHA-III-Symptomatik gelten als *nicht* fahrgeeignet [4, 5].

*Herzunterstützungssystem:* Nach individueller kardiologischer und kardiochirurgischer Beurteilung können Privatfahrer:innen auch mit Herzunterstützungssystem (z. B. Left Ventricular Assist Device – LVAD) als fahrgeeignet eingeschätzt werden [4, 5].

*Herztransplantation: *Bei herztransplantierten Patient:innen ist nach erfolgreicher Rehabilitation und klinisch stabilem Verlauf eine Fahreignung gegeben, sofern regelmäßige medizinische Kontrollen eingehalten werden [4, 5].

## Fahreignung bei Kardiomyopathien

Bei der *Kardiomyopathie* (Herzmuskelerkrankung) handelt es sich um einen Sammelbegriff für verschiedene Erkrankungen, die mit einem strukturell veränderten Herzmuskel einhergehen. Die Veränderungen des Herzens variieren abhängig von deren Ursache und können unter anderem zu einer krankhaften Verdickung der Herzwände, einer Erweiterung der Herzkammern, einer Abnahme der Herzleistung oder auch der Gefahr für Herzrhythmusstörungen bis hin zum PHT reichen. Einige dieser Veränderungen sind genetisch bedingt und betreffen somit Menschen auch schon in jungem Lebensalter. Die Vielzahl an möglichen Pathologien erklärt, warum keine einheitliche Aussage zur Fahreignung bei dem Vorliegen einer Kardiomyopathie getroffen werden kann. Die Prognose, stattgehabte Herzrhythmusstörungen sowie das Auftreten von Synkopen können aber im Einzelfall dazu führen, dass Privatfahrer:innen als nicht fahrgeeignet eingestuft werden [4].

Generell gilt jedoch auch hier, dass symptomfreie Patient:innen mit einer Kardiomyopathie ohne Herzrhythmusstörungen oder Synkopen in der Vergangenheit in der Regel als fahrgeeignet eingestuft werden [4, 5]. Es sollten allerdings regelmäßig fachärztliche Untersuchungen durchgeführt werden, um in Abhängigkeit von dem individuellen Krankheitsverlauf und unter Berücksichtigung von Beschwerden, Herzrhythmusstörungen und der einzuhaltenden Therapietreue eine individualisierte Einschätzung über die Fahreignung abgeben zu können. In einzelnen Fällen können validierte Risikokalkulatoren helfen, die Wahrscheinlichkeit für das Auftreten eines PHTs zu berechnen und ggf. die Implantation eines Defibrillators zu empfehlen [17, 18].

## Fazit

Die Fahreignung älterer Privatfahrer:innen stellt ein bedeutendes und praxisrelevantes Thema in der Kardiologie dar. Behandelnden Ärzt:innen stehen in vielen Situationen Richtlinien zur Einschätzung der Fahreignung aufgrund kardial bedingter Symptome oder Behandlungen zur Verfügung. Unabhängig von der Genese haben typische Symptome und die Wahrscheinlichkeit eines plötzlichen Bewusstseinsverlusts einen entscheidenden Einfluss auf die Beurteilung der Fahrtauglichkeit. Einschränkungen der Fahreignung bei Privatfahrer:innen aufgrund kardiovaskulärer Erkrankungen sind in Tab. 2 abschließend zusammengefasst. Da einige Pathologien nicht vollständig abgebildet sind und die Empfehlungen teils individuellen Behandlungsspielraum offenlassen, bleiben eine differenzierte Analyse sowie regelmäßige medizinische Untersuchungen von wesentlicher Bedeutung.

**Tab. 2:** Einschränkungen der Fahreignung aufgrund kardiovaskulärer Erkrankungen

Situation	Beschränkung der Fahreignung
*Koronare Herzkrankheit*	
Akutes Koronarsyndrom und LV-EF ≤ 35 %	In der Regel für 4 Wochen
Akutes Koronarsyndrom und kardiale Dekompensation	In der Regel für 4 Wochen
Koronare Bypass-Operation	In der Regel für 2–4 Wochen
*Arterielle Hypertonie*	
Symptomatische arterielle Hypertonie	Keine Fahreignung
*Herzklappenerkrankung*	
Symptomatische Herzklappenerkrankung (NYHA IV oder Synkope)	Keine Fahreignung
*Synkopen*	
Einmalige Synkope und Hinweise auf ein sehr hohes (Rezidiv‑)Risiko (s. oben)	Keine Fahreignung
Rezidivierende (unklare) Synkopen	Mindestens für 6 Monate
*Bradykardien*	
Unbehandelte Bradykardie mit Bewusstseinsverlust	Keine Fahreignung
Erworbener AV-Block Grad III	Keine Fahreignung
*Supraventrikuläre Tachykardien*	
Unbehandelte Tachykardie mit Bewusstseinsverlust	Keine Fahreignung
*Ventrikuläre Tachykardien*	
Unbehandelte ventrikuläre Tachykardie (> 30 s)	Keine Fahreignung
Überlebter PHT (ohne ICD-Versorgung) jeglicher Genese	Keine Fahreignung
*Schrittmacher/Defibrillator (ICD)*	
Nach Schrittmacher‑/ICD-Aggregatwechsel oder Sondenwechsel sowie primärprophylaktischer ICD-Implantation	Für 1–2 Wochen
Sekundärprophylaktische ICD-Implantation	Mindestens für 3 Monate
Nach adäquatem ICD-Schock	Mindestens für 3 Monate
Nach inadäquatem ICD-Schock	Keine Fahreignung bis zur Behebung der Ursache
Elektrischer Sturm/rezidivierende sVT unter ICD-Versorgung	Einzelfallentscheidung; mindestens für 3 Monate
*Ionenkanalerkrankungen*	
Long-QT-Syndrom/BrS mit Synkopen, QTc-Zeit >500 ms oder Torsaden	Keine Fahreignung
*Herzinsuffizienz*	
Herzinsuffizienz der Klasse IV oder bei instabiler NYHA III	Keine Fahreignung

*AV-Block* atrioventrikulärer Block, *BrS* Brugada-Syndrom, *ICD* implantierbarer Kardioverter-Defibrillator, *LV-EF* linksventrikuläre Ejektionsfraktion, *NYHA* New York Heart Association, *PHT* plötzlicher Herztod, *sVT* anhaltende ventrikuläre Tachykardie
